# SDN Control Strategy and QoS Optimization Simulation Performance Based on Improved Algorithm

**DOI:** 10.1155/2022/7167957

**Published:** 2022-03-18

**Authors:** Bin Zhang, Xin Liu

**Affiliations:** School of Information Engineering, Shandong Youth University of Political Science, Jinan, Shandong 250103, China

## Abstract

In the past few years, the Internet has become more and more popular, and more and more people use the Internet. A lot of new software has been developed on the Internet, so the network traffic has increased rapidly. A company once clearly stated that, in the future, the network volume in our country will reach nearly 5 zb. In addition, the network is comprehensively unified, and the learning of the Internet is strengthened by the way of suggesting errors. It is also necessary to learn from the external environment to make the finite value of the network as good as possible. Reinforcement learning is about solving many difficult problems. Use reinforcement learning to promote and change this state, so that the advantages of the network can be fully developed. Regarding the environment and actions at the time as the basic mapping requirements, and the network technology strategy can be best developed. In order to solve the QoS optimization scheme of the current mainstream heuristic algorithm in the software-defined network scene, the software-defined network QoS optimization algorithm is proposed. First, the network resources and status information are unified into the network model, and then, the long- and short-term memory network is used to improve the algorithm's flow perception ability. Finally, based on the deep reinforcement learning algorithm, a dynamic traffic scheduling strategy that satisfies the QoS objective is constructed. Among them, QoS refers to the main functions of the entire network system, especially for some new users, but for many old users, this system can represent a certain application, for example, whether a certain software can get timely, whether it can work smoothly when processing videos, and whether it can be interrupted when making a voice call for improvement.

## 1. Introduction

In the upcoming 2022, the total amount of China's network will reach 5zb. This number represents that the Internet is being widely used, and basically the Chinese are using the Internet from door to door [1]. It also proves that the number of people using the Internet is innumerable. Faced with the rapid development of the Internet, on the premise that many large companies and consumers use the Internet at the same time, Internet companies are also undergoing an unprecedented huge test [2]. This is because when many consumers use the same network at the same time, problems such as freezes, unclear videos, and unusable traffic will occur [3]. At present, the basic meaning of software-defined networking is to accept the major changes of the newly developed network [4]. Because the original network also has some problems, we have to find many new ways to solve these original problems. The basic purpose of the software is to unbind the initial network control, separate it from the original, and then use the control panel to perform a comprehensive search, so that our program will not be stuck or unclear status [5]. This program was then sent to the company above, in order to unify the network in an all-round way, so as to facilitate the management of the staff and make the network develop in an orderly manner [6]. The main reason is to make network management simple because many people still do not know how to use the network [7]. If it is too difficult, they will face many problems. After solving the difficult problems, the cost must be minimized, so that our costs are reduced, and the highest benefits can be obtained, and consumers can use it with confidence [8]. We must also greatly support the advantages of network innovation and then find out many shortcomings and solve them one by one [9]. There are many large and well-known Internet companies that are also applying this feature to give full play to their advantages, learn from each other's strengths, and maximize the interests of consumers [10]. This can also dig out potential functions on the largest scale and achieve utilization. The biggest is to change some of the functions of the network and to properly solve the previous bad aspects, so that we can use it comfortably. The goal is to make the network more meaningful, and the meaning of the network itself lies in letting consumers solve problems, make their lives more convenient, and meet the interests of consumers [11]. Only when the Internet is done can our lives become more colorful and beautiful.

## 2. Related Work

Literature [12] proposes that there is a lot of false information on the Internet, because the Internet itself is virtual, and many things you see may not necessarily mean that it is real, because some consumers do not understand this aspect. At the same time, many criminals have processed network information, so that many consumers have been deceived by false information on the Internet. To solve this difficult problem, the relevant departments have come up with a solution. This method is used to connect the Internet and resources. The hub of the Internet is to solve the virtual existence of the Internet, help consumers not be deceived, and strengthen online learning. Literature [13] concisely put forward some problems existing in the network and based on these existing problems came up with a solution and then constructed a model, which is called a mathematical model. Literature [14] provides a mechanism that can manage and solve this problem well. In Literature [15], it is pointed out that because the user's choice is different, each solution is different, and the strategy is also different. Literature [16] has good control ability and good calculation ability, so the content designed according to its characteristics is different, and the network path and change system are also different. Literature [17] chose to introduce a new type of technology, which can change the original resource and network allocation. In the literature [18], there is a model that proves that the use of management methods can solve the problems well. Literature [19] uses it to link with broadband in a wireless network. With broadband, half of the problem can be solved in this way. Literature [20] pointed out that there are many places where the network is used, and many places have problems that are difficult to solve. Aiming at this series of problems, an organization can solve the current problems, and its name is called the regional network. Because each piece of network is divided into different regions, each region has its own network, which solves some of them well. And because the network sometimes has disconnection problems, problems like freezing of videos occur. Literature [21] proposed software that can use algorithms to control the virtuality of the network and the allocation of resources and the network. The ultimate goal is to solve the problem of insufficient allocation. Literature [22] applies governance technology to the network and proposes two solutions. Literature [23] pointed out that the use of open data to manage the traffic required in the network will solve the problem of unclear video network stalls. It also proposed a document mechanism to further ensure the high quality of the video and a better quality of the traffic network in the increasing number of traffic videos and the most basic difference, so as to meet the needs of consumers to a greater extent. A good network and network companies are also improving, and our lives are also progressing and will become better.

## 3. SDN Network Analysis Based on Deep Reinforcement Learning

### 3.1. SDN Control Strategy Based on Deep Reinforcement Learning

Reinforcement learning is to use an intelligent method to learn, using the environment as a guide for the next step, and its ultimate goal is to better reflect the intelligence and maximize the benefits. Through reinforcement learning, many ways to solve the problem have been discovered. One of them is because there is little information obtained through the external environment, and it must rely on its own experience and original materials to learn, and it has obtained it in the subsequent environment. Reinforcement learning can obtain a lot of information from the environment, so as to analyze and change the current environment. There is an algorithm that can be used as a major point of solving the problem, and that is to calculate the resource allocation in the wireless network. This calculation should be solved first. In the algorithm, we have to make analogies and assumptions and then calculate the number of calculations. The results are calculated, and the △ is the most important core. Observe and get rewards or punishments. The following are rewards or punishments:

(1) $$\begin{array}{c} {\text{R}}_{\text{t}}=\sum_{\text{i}=\text{t}}^{\text{T}}\gamma^{\left({\text{t}}^{\prime }-\text{t}\right)}\cdot \gamma_{\text{i}}. \end{array}$$

Among them, *y* belongs to a factor, and *R* is calculated from beginning to end and includes the sum of rewards and the sum of punishments. In addition, *t* means time and *q* is defined as a special dynamic variable in mathematics. The core of an algorithm is to use formulas for algebra. Use this method to learn and use simpler methods to calculate dynamic variables:

(2) $$\begin{array}{c} \left\{\begin{array}{c} {\text{Q}}_{\text{k}+1}{\text{s}}_{\text{t}},\alpha_{\text{t}}={\text{Q}}_{\text{k}}{\text{s}}_{\text{t}},\alpha_{\text{t}}+\alpha_{\text{k}}\cdot \delta_{\text{k}},\\ \delta_{\text{k}}=\gamma_{\text{t}+1}+\gamma \cdot {\text{maxQ}}_{\text{k}}{\text{s}}_{\text{t}+1},{\text{a}}^{\prime }-{\text{Q}}_{\text{k}}{\text{s}}_{\text{t}},\alpha_{\text{t}}\left({\text{a}}^{\prime }\in A\right). \end{array}\right. \end{array}$$

In the formula, *α* represents the time and rate of learning. In addition, *s* and a, respectively, represent the distance and a certain state of the action, which is the difference in time, and *α* represents the state and distance of the execution *n* times. In addition, in order to realize the algorithm of formula two, the conditions must be met:

(3) $$\begin{array}{c} \left\{\begin{array}{c} \sum_{\text{k}}{\text{a}}_{k}^{2}＜+\infty ，\sum_{\text{k}}\alpha_{\text{k}}=+\infty ,\\ \underset{\text{k}⟶\infty }{\text{lim}}{\text{Q}}_{\text{k}}={\text{Q}}^{*}. \end{array}\right. \end{array}$$

According to the process described above, we can know that in a certain state, and we can choose the best method by ourselves; that is, choose the best expected value to analyze a specific function:

(4) $$\begin{array}{c} {\text{Q}}^{*}\text{s},\text{a}={\text{E}}_{{\text{s}}^{\prime }-\xi }\left(\text{r}+\gamma \cdot \text{max}\right){\text{Q}}^{*}\left({\text{s}}^{\prime },{\text{a}}^{\prime }|\text{s},\text{a}\right). \end{array}$$

In the general environment, various states will exist. *s* is regarded as a certain state after performing an action, and *α* is a combination of one state and another state and executed.

#### 3.1.1. Constructing Special Value Functions

By strengthening the learning of the special value function in the network, taking this function as the core problem in transforming it into another function, it can be expressed as

(5) $$\begin{array}{c} \hat{\text{Q}}{\text{s}}_{\text{sys}},\text{a};\Theta \approx {\text{Q}}^{\pi }\left({\text{s}}_{\text{sys}},\text{a}\right). \end{array}$$

In a more in-depth research algorithm, if you want to calculate a certain function, you can rely on a model. This model can update itself and then calculate it. The special value function in the Q-Learning algorithm can be rewritten as

(6) $$\begin{array}{c} {\text{Q}}^{*}{\text{s}}_{\text{sys}},\text{a}={\text{Qs}}_{\text{sys}},\text{a}+\text{a}\left[{\text{R}}_{\text{once}}+{\gamma \text{maxQs}}_{\text{sys}},{\text{a}}^{\prime }-\text{Q}\left({\text{s}}_{\text{sys}},\text{a}\right)\right]. \end{array}$$

Among them, a is a learning factor, and a is a learning factor in another state and a certain action in another state.

A certain kind of table cannot store every state of the system, so we have to change this kind of table and solve the solution in this article, which is to let the functions replace each other and express them by approximate functions.

#### 3.1.2. Constructing Loss Function under Dual Network Architecture

To make the results calculated by this algorithm more specific, two very similar network mechanisms must be constructed. One of the mechanisms is relatively stable and does not change frequently. We call it the destination network and use a letter to value it. Another mechanism is called the core network. Determine a certain function by strengthening the computing power, and its loss function expression is

(7) $$\begin{array}{c} \underset{\text{k}⟶\infty }{\text{lim}}{\text{Q}}_{\text{k}}=\Theta_{*}. \end{array}$$

A function gradient can be obtained by calculating through listing:

(8) $$\begin{array}{c} \frac{\text{dL}\Theta }{\text{d}\Theta }=\text{E}\left[{\text{Q}}_{\text{target}}-\left({\text{s}}_{\text{sys}},\text{a};\Theta \right)\frac{\text{dQ}\left({\text{s}}_{\text{sys}},\text{a};\Theta \right)}{\text{d}\theta }\right]. \end{array}$$

Use the previously mentioned formula to calculate the function gradient for a lot of training and finally get the most accurate function value, and the purpose is to achieve the goal of special points. There is a special derivative:

(9) $$\begin{array}{c} \nabla_{\Theta_{\text{k}}}{\text{L}}_{\text{k}}\Theta_{\text{k}}=\text{E}\left[\left({\text{R}}_{\text{once}}+{\;\gamma \text{maxQ}}_{\text{main}}{\text{s}}_{\text{sys}}^{\prime },{\text{a}}^{\prime }；\Theta_{\text{k}-1}-{\text{Q}}_{\text{main}}{\text{s}}_{\text{sys}},\text{a};\Theta_{\text{k}}\right)\nabla_{\Theta_{\text{k}}}{\text{Qs}}_{\text{sys}},\text{a};\Theta_{\text{k}}\right]. \end{array}$$

Among them, the value of one parameter is fixed.

#### 3.1.3. Experience Playback

(10) $$\begin{array}{c} {\text{Qs}}_{\text{sys}},\text{a};\Theta_{\text{k}}\overset{\text{modification}}{⟶}\text{Q}\left({\psi \text{s}}_{\text{sys}}，\text{a},\theta^{\prime }\right). \end{array}$$

In addition, an input state in the ΦBei network system is also a combination of two parameters including neural network and destination network. The corresponding mechanism can be modified to

(11) $$\begin{array}{c} \left\{\begin{array}{c} D\overset{\text{modification}}{⟶}\bar{\text{D}}=\left[\bar{{\text{e}}_{1},}\bar{{\text{e}}_{2}},\ldots \bar{{\text{e}}_{\text{l}}}\right],\\ \bar{{\text{e}}_{\text{i}}}=\left(\psi {\text{s}}_{\text{sys},\text{i}},{\text{a}}_{\text{net},\text{i}},{\text{r}}_{\text{once},\text{i}},\psi {\text{s}}_{\text{sys},\text{i}+1}\right). \end{array}\right. \end{array}$$

### 3.2. Analysis of SDN Framework Modules

#### 3.2.1. QoS Routing

*Topology Management Module.* There is a module that uses an automatic check for network problems to identify the connection between the controller and the noncontroller switch and the switch and automatically write down the Internet address, IP address, and open data and other information, and check information: save, delete, update and change regularly.

*Network Monitoring Module.* One reaction in the network is to block the network traffic. The more serious the blockage is, the stall will occur. Use (12) to calculate the link congestion rate:

(12) $$\begin{array}{c} {\text{g}}_{\text{link}}=\frac{C}{\text{B}}. \end{array}$$

In Table 1, B represents the amount of stutter in the video, C represents the amount of bandwidth in the link, and *g* represents the sum between the two quantities. Therefore, if we want to calculate this number, we must find the calculation number in it. The China map finds that it can be represented by the amount of special points and the amount of broadband at special points. The acquisition of the open protocol is simple, and it can be obtained by receiving and sending through the port. If you want to obtain the flow table, you must also use the calculation shown in Table 1.

When performing statistics, you can only select the port and number of special points, the number of bytes, and the time included. At *t*_1_, the transmission of a special point is represented by a letter, and the number of bytes received is R. At time *t*_2_, the transmission of a special point is represented by another letter, which represents a received time, and finally find the corresponding result. The finally obtained network monitoring data can be expressed by Equation (16):

(13) $$\begin{array}{c} \text{B}=\frac{{\text{Tx}}_{2}-{\text{Tx}}_{1}}{{\text{t}}_{2}-{\text{t}}_{1}}+\frac{{\text{Rx}}_{2}-{\text{Rx}}_{1}}{{\text{t}}_{2}-{\text{t}}_{1}}. \end{array}$$

We can also calculate the result we want using formulas (15) and (16).

The open protocol does not have a way to directly detect the network delay, so it is not a good method to use the open protocol to calculate the delay.

*Path Calculation Module*. Use the methods passed through to calculate the comprehensive network information and use the data provided by the network monitoring module. Relatively speaking, the highest-level data is used for statistics, and other low-level data should be used with the least. The path is calculated at the cost.

*Routing Management Module*. By controlling the router to calculate the number we want and send it to the company for the company to carry out the next exchange, the switch can carry out the next business forwarding. It mainly contains the following functions:

This module can identify different businesses differently. After identifying the highest and best data, it will be sent to other companies and the results will be calculated. Compared with other levels of data, the results will be sent out. The best result can be calculated at the cost of the shortest path.

#### 3.2.2. Queue Scheduling Management

*Queue Agent Module*. In a modular display controller, if we want to open the company to exchange and communicate commands, this is what we want.

*Traffic Management Module*. The flow module is used for management in the open switch, so that the rapid channel and the template in the controller can be integrated with each other. The related queue scheduling and simple algorithm become very simple, and the management strategy can be analyzed and answered later.

### 3.3. Analysis and Design of Lagrangian Relaxation Routing Algorithm

#### 3.3.1. Delay-Constrained Minimum Cost Path Problem

There are definitely the most advanced and advanced ones in the network, and there are also some problems with delay and congestion in the network. If you want to find this value, you want to minimize link congestion. Then, we have to figure out a way to solve it.

(14) $$\begin{array}{c} \text{cp}=\sum_{\text{u},\text{v}\in \text{p}}{\text{c}}_{\text{uv}}\\ \text{dp}=\sum_{\text{u},\text{v}\in \text{p}}{\text{d}}_{\text{uv}}\\ {\text{p}}^{*}=\text{min}\left\{\text{cp}|\text{dp}\leq \Delta_{\text{delay}}，\text{p}\in {\text{P}}_{\text{st}}\right\}. \end{array}$$

#### 3.3.2. Using Lagrange's Median Theorem for Algebraic Algorithms

In real life, there are good ways to solve this problem, but it is impossible to find a theory to replace this algorithm. It is necessary to use Lagrange's median value theorem to approximate this value, so that this problem can be solved.

LARAC is implemented by an aggregate cost function, which is shown in formulas (15) and (16).

(15) $$\begin{array}{c} {\text{c}}_{\text{uv}}\lambda ={\text{c}}_{\text{uv}}+\lambda \times {\text{d}}_{\text{uv}}, \end{array}$$

(16) $$\begin{array}{c} {\text{c}}_{\lambda }\text{p}=\sum_{\text{u},\text{v}\in \text{p}}{\text{c}}_{\text{uv}}\lambda . \end{array}$$

Use a letter to represent Lagrange's median theorem, and *c* represents a level of aggregate cost function.

Use Lagrange's median theorem to calculate link congestion rate and weighted rate; see equation (18).

(17) $$\begin{array}{c} {\text{c}}_{\text{uv}}={\text{ag}}_{\text{uv}}+1-\text{a}\times 1,0\leq \text{a}\leq 1，\forall \text{u},\text{v}\in \text{E,} \end{array}$$

(18) $$\begin{array}{c} \lambda =\frac{\text{c}\left({\text{p}}_{\text{c}}\right)-\text{c}\left({\text{p}}_{\text{d}}\right)}{\text{d}\left({\text{p}}_{\text{d}}\right)-\text{d}\left({\text{p}}_{\text{c}}\right)}. \end{array}$$

If d(pr)≤∆delay, then a new way must be found; otherwise, the solution cannot be solved by using that way.

### 3.4. PDW Queue Scheduling Management Algorithm

According to a technique for randomly detecting queues, this technique can be divided into two algorithms: one algorithm is to calculate the average queue length, and the other algorithm is to calculate the probability. The detailed calculation formula is shown as follows:

(19) $$\begin{array}{c} {\text{q}}_{\text{ave}}{\text{t}}_{\text{n}}={\text{wq}}_{\text{ave}}{\text{t}}_{\text{n}-1}+\left(1-\text{w}\right){\text{qt}}_{\text{n}}. \end{array}$$

The probability of packet discarding is related to the minimum value min, maximum value max, and the reference probability *p*. The calculation formula for the discarding probability *p* is shown as follows:

(20) $$\begin{array}{c} \text{p}={\text{p}}_{\text{b}}\times \frac{\left.{\text{q}}_{\text{ave}}{\text{t}}_{\text{n}}-{\text{q}}_{\text{th}\_\text{min}}\right)}{{\text{q}}_{\text{th}\_\text{max}}-{\text{q}}_{\text{th}\_\text{min}}}. \end{array}$$

This algorithm is the biggest change to the previous algorithm, and it can judge the level of scheduling management and carry out the congestion processing rate. And its change is to add a level-related processing on the original basis. And through this kind of relevant processing, it is very good to improve the service quality of the network.

## 4. Software-Defined Network QoS Optimization and Implementation

### 4.1. QoS Control Framework and Route Optimization

**Figure 1:**
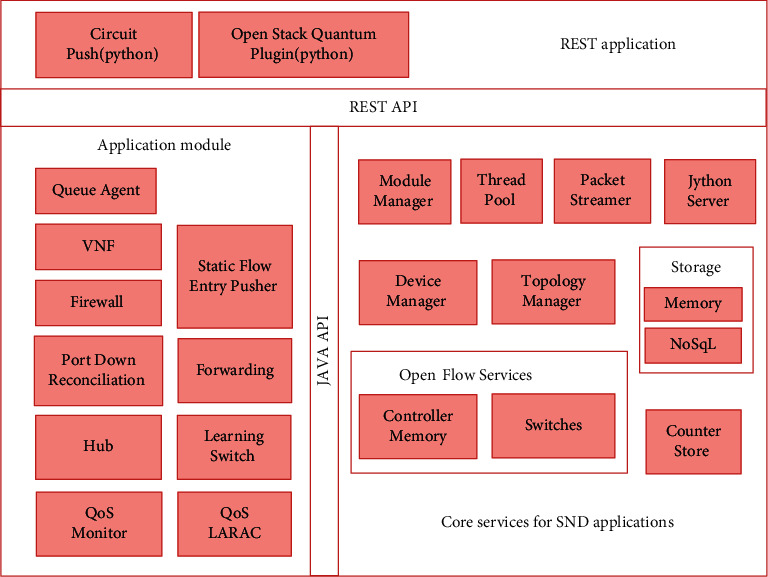
Prioritized QoS control is implemented in Floodlight.

The router involves four modules: network management module, network monitoring module, network path calculation module, and routing management module, as shown in Figure 1.

#### 4.1.1. Topology Management Module

As soon as the open protocol enters a certain network mechanism, the controller will respond quickly and discover the protocol and periodically control the machine and switch to exchange information with the switch and then change and update these links. The last step is that the exchange events of these links are recorded in the list.

This kind of controller uses the shortest path for calculation, and its disadvantage is that it cannot make advanced distinctions based on the shape of the network. It can also protect all kinds of information on the network from being infringed. It can also use a certain method to exchange and protect the mapping information of the node based on the comprehensive mapping information provided by the network monitoring module. The purpose of this method is to map the switch node to other switch nodes. This method is also the realization of the algorithm. After the switches are exchanged, two methods can be obtained to obtain the minimum path between the switches.

**Figure 2:**
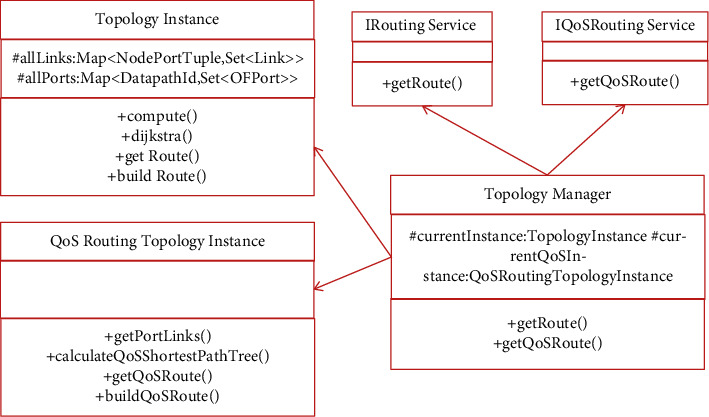
The relationship between related classes and interfaces of topology management module.

This kind of management system will make periodic changes, change the information according to this periodic change, and then perform different corresponding processing based on this information. The purpose is to protect the stability of this network. This kind of management system also has very important components such as top management. With this component, the shortest path and minimum cost between special points can be calculated. The relationship between related classes and interfaces of topology management module is shown in Figure 2.

#### 4.1.2. Network Monitoring Module

The network monitoring module can obtain comprehensive data and network congestion rate periodically and orderly and calculate the path to maximize the processing of this information. Among the other two modules, the two network monitoring modules are also a good choice to calculate data in an orderly manner.

Module management should be performed on the link, and the controller should be used to expand the module, and the control module should be used as the most basic management module. Let other modules forward and discard the processing separately. The most important module is processing. After processing, it can be forwarded according to the path.  Table 2 contains the main methods.

### 4.2. Experimental Network Test Platform

#### 4.2.1. Experimental Network

The software and hardware configuration of each node in the network is shown in Table 3. In the table, DPID/IP represents the switch network identification number or the terminal host IP address.

#### 4.2.2. Business Priority Mark

To distinguish different services differently, it is necessary to carry out a special mark for each type of service. From the following table, we can know that there are many special values for the business priority level of the business type, as shown in Table 4.

#### 4.2.3. Experimental Testing Tools

Iperf is a network performance testing tool that can test the network data reception rate and packet loss rate. Use this software to send and receive data, and test the data receiving rate and packet loss rate.

### 4.3. QoS Optimization Function Test and Result Analysis

#### 4.3.1. Experiment 1: QoS Path Calculation Validity Test

- Experimental purpose
- Under the premise of network congestion, the calculation method is used to find the result with a small path cost.
- Experimental scenarios and steps
- Use the HTB queue in LinuxTC to limit the maximum sending rate of the switch port to 5 Mbps. Since the network mainly transmits UDP packets, the transmission volume of other types of packets is small, so the maximum transmission rate of the switch port is used as the bandwidth of the link connecting the port.
- Experimental results and analysis

According to Table 5, we can know that if the congestion rate is different, its result will be different.

**Figure 3:**
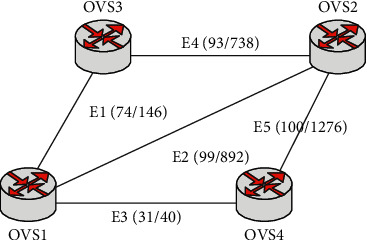
QoS routing topology.

From Table 5, we can see how fast the router is, its congestion rate, its cost, and some important information. Its supply chain is shown in Figure 3. It can be seen that the data in parentheses can reflect the price of the cost, which can be obtained by calculation. So this router is composed of OVS4 and OVS1, so that the cost can be lower.

#### 4.3.2. Experiment 2: Dynamic Rerouting Effectiveness Test

① Experimental purpose
(i) When the network QoS path is congested, the path can no longer meet the QoS requirements of the highest priority service data flow. It is necessary to test whether the highest priority service data flow can be rerouted to the new QoS path when the QoS path is congested.
  ② Experimental scenarios and steps
(ii) In experiment 1, the QoS transmission path is OVS4- > OVS2- > OVS3- > OVS1, and the Floodlight controller will store this QoS path in the path set cache. After experiment one, continue with this experiment. The experimental steps are as follows:
  1. On the switch side, use the flow table delete command to delete the flow table entries in the switch.
  2. Add static flow entries in OVS4, OVS2, and OVS1, so that HOST5 sends background UDP packets with the transport layer source port number of 25551 to HOST6 through these three switches in turn.
  3. HOST5 sends UDP messages with source port numbers of 25554 and 25551 to HOST6, and the messages are sent for 30 minutes. Since the Floodlight controller path set caches the session message QoS path, the session message transmission path is OVS4- > OVS2- > OVS3- > OVS1, and the background message transmission path is OVS4- > OVS2- > OVS1.
  4. Read Floodlight log, obtain network link parameters and update QoS path.
  ③ Experimental results and analysis

Looking at Table 3, we can see that Floodlight calculates the QoS path as OVS4- > OVS1.

From the data in Table 6, it can reflect some accidents, costs, and important information encountered when the router is in use. When we are using the LARAC algorithm, we can calculate the final result. The information we get from now is the speed of the router, how much it costs, and how long it takes to get the network link without crowding it. It has the best service, that is, it can pass QoS, so that we can know whether the path will be updated by comparing the old and new data.

### 4.4. QoS Optimization Performance Test and Result Analysis

#### 4.4.1. Experiment 3: Dispatching and Managing the Network and Inspecting Its Performance

**Figure 4:**
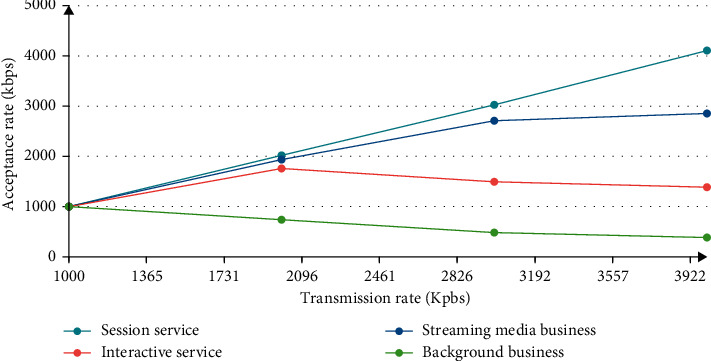
PDW policy classification service receiving rate.

**Figure 5:**
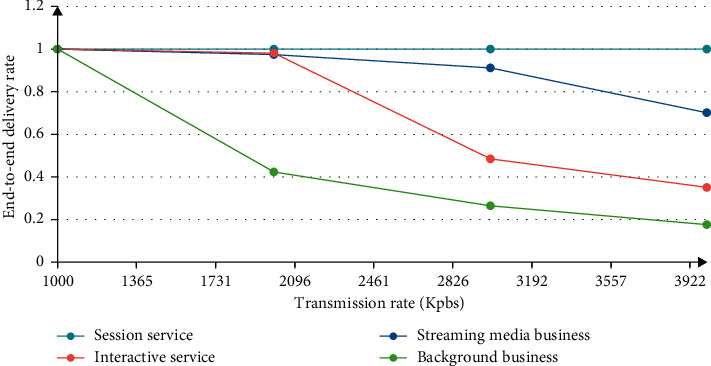
PDW strategy classification business submission rate.

The higher the level, the faster the speed; the QoS performance will be best protected, which can explain the importance of performance and speed, as shown in Figures 4-5.

The higher the level, the better the effect, and the performance of QoS can be protected in advance. This explains the requirement that the PDW policy distinguishes performance and protects different levels of performance.

#### 4.4.2. Experiment 5: Control Frame Level and Performance Test

(i) ① Experimental purpose
  In this experiment, we can obtain important information about the speed of reception and transmission. Only after this operation can we detect the benefits of framework performance.
  ② Experimental scenarios and steps

We can understand from the figure, the importance of broadband transmission speed. The broadband performance is good, the speed is fast, the control frame level is higher, and at the same time, a high-performance processor is required to operate, so that there will be no delay.

**Figure 6:**
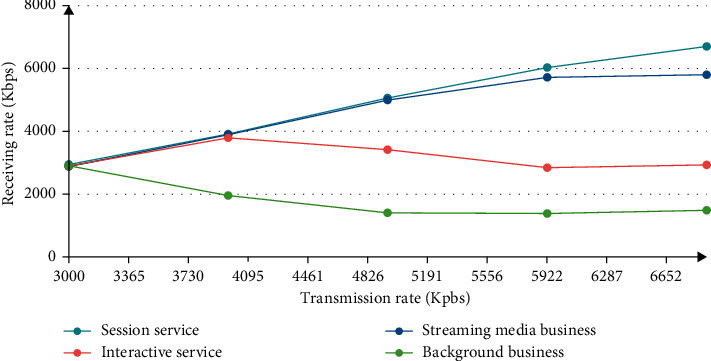
QoS control framework receiving speed of different priority packets.

**Figure 7:**
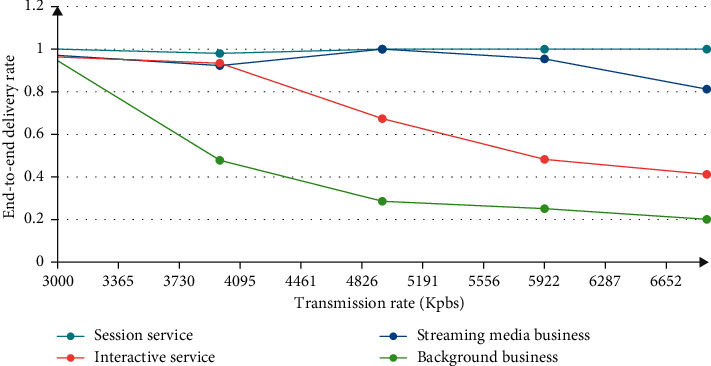
QoS control framework different priority message delivery rate.

When we look at Figures 6 and 7, we can see at a glance the usage of the sending rate and the importance of the framework.

Figure 7 shows how fast the best sending rate is, and the most advanced receiving speed can better stabilize this program. When the sending rate reaches 2 times, the data transfer speed will be very fast, and the program can be stabilized without the best telegram. Then, its stability needs a best suit to complete, making it more advanced. From this point of view, the higher the QoS framework, the better, and the QoS control framework also achieves better stability, improves network utilization, increases the amount of data transmission, and improves usage efficiency, so it can be used better.

## 5. Conclusion

We all know that, in today's networks, the speed of the network is fast or slow; some are very fast, and some are very slow, and even disconnected and stuck. And the quality is good or bad. Some networks are of good quality and very efficient, and some of them are not satisfactory, so we need to strengthen network learning. The speed of the network speed and the quality of the network are important factors that affect the network, and the most advanced transmission and reception are guaranteed, and other services have received the best service from then on. This is the network we need most. In the network, we also need to study the priority levels of different services. For the most advanced clients, we must ensure timely solutions. For some low-level services, we must perform module partition processing. Research the QoS of different priority services in the SDN network, provide end-to-end delay guarantee for the highest priority services, and provide differentiated QoS services for other services. By introducing the concept of QoS, two traditional network models that can improve QoS are explained, and the characteristics of the models are analyzed. The four types of services transmitted in the network are differentiated and set according to their QoS requirements. In order to ensure the QoS of different services, a QoS control framework in the SDN network is proposed. The various functional modules in the QoS control framework are implemented in Floodlight and OpenvSwitch soft switches. From the previously mentioned situation, the QoS framework has important functions and advantages. It can make the speed faster, the quality better, and it also promotes better service. It has been welcomed by the public, so the development of routers is a good prospect. There will be better development in the future, which can help people observe and receive information anytime and anywhere, but also the utilization rate has been well developed, without polluting the environment, and expanding the transmission volume. At the same time, it is also necessary to know how to develop a better framework and exchange tests based on this original QoS control framework, as well as the control of the control framework by the switches in the network. The switch plays a very important role in the network because it can make the best use of the various functions in the framework and finally become a reality, so that some false things in the network no longer exist and the network can function as a router. Performing a perfect test to maximize the benefits of the network is the best result we need.

## Figures and Tables

**Table 1:** PORT_STATS_REPLY traffic statistics structure.

structofp_port_stats{	
uint16_tport_no;	/ *∗* The port number*∗*/
uint64_trx_packets;	/ *∗* Number of received messages*∗*/
uint64_trx_packets;	/ *∗* Number of sent messages*∗*/
uint64_trx_bytes;	/ *∗* Number of bytes received*∗*/
uint64_trx_bytes;	/ *∗* Send bytes*∗*/
uint64_trx_bytes;	/ *∗* Duration*∗*/
};	

**Table 2:** Main methods of DynamicQoSRoute class.

Method	
addPath	The source switch destination switch pair is used as an identifier, a new QoS path is added to the path set cache, and the increased path time is stored at the same time.
updatePath	Update the QoS path between the specific source switch and destination switch pair in the path cache.
delPath	Delete the timeout invalid path in the path set cache.

**Table 3:** Network node software and hardware configuration.

Node	Hardware	Software system	DPID/IP
HOST1	Dell-OPTIPLEX-360	Ubuntu-12.04(linux-3.13.1)	10.0.3.2/24
HOST2			10.0.3.4/24
HOST3			10.0.3.5/24

HOST4			10.0.3.6/24
HOST5			10.0.3.22/24
HOST6			10.0.3.44/24
OVS1		Ubuntu-12.04(linux-3.13.1) OpenvSwtich-2.3.0	00 : 00 : 00:0e:c6:c1:56:dc
OVS2			00 : 01 : 02 : 03 : 04 : 05 : 06 : 07
OVS3			00 : 00 : 00:0e:c6:cb:3b:5 b
OVS4			00 : 00 : 00:0e:c6:c1:f4:1c

F1	Dell-OPTIPLEX-9020	Ubuntu-12.04(linux-3.13.1) Floodlight1.2	—

**Table 4:** Business priority mapping.

Business type	Business priority value	Transport layer source port number	Ds domain value	Tos field value
Conversational business	4	25554	4	16
Streaming business	3	25553	3	12
Interactive business	2	25552	2	8
Background business	1	25551	1	4

**Table 5:** Network link congestion rate and delay information.

Link	Congestion rate	Cost price	Time delay (ms)
E1(OVS3- > OVS1)	0.1727	26	20
E2(OVS2- > OVS1)	0.4096	46	60
E3(OVS4- > OVS1)	0.8259	84	145
E4(OVS2- > OVS3)	0.3254	39	28
E5(OVS4- > OVS2)	0.1067	19	15

**Table 6:** Update the link congestion rate and delay information before the QoS path.

Link	Congestion rate	Cost price	Time delay (ms)
E1(OVS3- > OVS1)	0.7145	74	146
E2(OVS2- > OVS1)	0.9925	99	892
E3(OVS4- > OVS1)	0.2317	31	40
E4(OVS2- > OVS3)	0.9252	93	738
E5(OVS4- > OVS2)	0.9999	100	1276

## Data Availability

The data used to support the findings of this study are included within the article.

## References

[1] Zhang Q., Cheng L., Boutaba R. (2010). Cloud computing: state-of-the-art and research challenges. *Journal of Internet Services and Applications*.

[2] Shi W., Cao J., Zhang Q., Li Y., Xu L. (2016). Edge computing: vision and challenges. *IEEE Internet of Things Journal*.

[3] Lai P., He Q., Cui G. Edge user allocation with dynamic quality of service. *Service-Oriented Computing*.

[4] Blenk A., Basta A., Reisslein M., Kellerer W. (2016). Survey on network virtualization hypervisors for software defined networking. *IEEE Communications Surveys & Tutorials*.

[5] Thyagaturu A. S., Mercian A., McGarry M. P., Reisslein M., Kellerer W. (2016). Software defined optical networks (SDONs): a comprehensive survey. *IEEE Communications Surveys & Tutorials*.

[6] Vilalta R., López V., Mayoral A. The need for a Control Orchestration Protocol in research projects on optical networking.

[7] Chowdhury N. M. M. K., Boutaba R. (2010). A survey of network virtualization. *Computer Networks*.

[8] Lam K., Davis N. (2015). Open networking foundation, technical recommendations ONF TR-512. *Core information model (coremodel)*.

[9] Iovanna P., Cavaliere F., Testa F. (2016). Future proof optical network infrastructure for 5G transport. *Journal of Optical Communications and Networking*.

[10] Schönwälder J., Björklund M., Shafer P. (2010). Network configuration management using NETCONF and YANG. *IEEE Communications Magazine*.

[11] Initiative 5G. (2015). *5g white Paper,” NGMN Alliance, Final Deliverable 1.0*.

[12] Molina M. D., Sundar S. S., Le T., Lee D. (2021). Fake news is not simply false information: a concept explication and taxonomy of online content. *American Behavioral Scientist*.

[13] Shu K., Mahudeswaran D., Wang S., Lee D., Liu H. (2020). FakeNewsNet: a data repository with news content, social context, and spatiotemporal information for studying fake news on social media. *Big Data*.

[14] Jeon K. E., She J., Soonsawad P., Ng P. C. (2018). BLE beacons for internet of things applications: survey, challenges, and opportunities. *IEEE Internet of Things Journal*.

[15] Wei W., Ke Q., Nowak J., Korytkowski M., Scherer R., Woźniak M. (2020). Accurate and fast URL phishing detector: a convolutional neural network approach. *Computer Networks*.

[16] Santoso H. A., Rachmawanto E. H., Nugraha A., Nugroho A. A., Rosal Ignatius Moses Setiadi D., Basuki R. S. (2020). Hoax classification and sentiment analysis of Indonesian news using Naive Bayes optimization. *TELKOMNIKA (Telecommunication Computing Electronics and Control)*.

[17] Mahapatra S. N., Singh B. K., Kumar V. (2020). A survey on secure transmission in internet of things: taxonomy, recent techniques, research requirements, and challenges. *Arabian Journal for Science and Engineering*.

[18] AlZu’bi S., Hawashin B., Mujahed M. (2019). An efficient employment of internet of multimedia things in smart and future agriculture. *Multimedia Tools and Applications*.

[19] Nica E., Janoškova K., Kovacova M. (2020). Smart connected sensors, industrial big data, and real-time process monitoring in cyber-physical system-based manufacturing. *Journal of Self-Governance and Management Economics*.

[20] Sankar S. P., Subash T. D., Vishwanath N., Geroge D. E. (2021). Security improvement in block chain technique enabled peer to peer network for beyond 5G and internet of things. *Peer-to-Peer Networking and Applications*.

[21] Li J., Maiti A., Springer M., Gray T. (2020). Blockchain for supply chain quality management: challenges and opportunities in context of open manufacturing and industrial internet of things. *International Journal of Computer Integrated Manufacturing*.

[22] Shrestha Y. R., Ben-Menahem S. M., Von Krogh G. (2019). Organizational decision-making structures in the age of artificial intelligence. *California Management Review*.

[23] Keane E., Zvarikova K., Rowland Z. (2020). Cognitive automation, big data-driven manufacturing, and sustainable industrial value creation in internet of things-based real-time production logistics. *Economics, Management, and Financial Markets*.

